# Clinical Potential of Misshapen/NIKs-Related Kinase (MINK) 1—A Many-Sided Element of Cell Physiology and Pathology

**DOI:** 10.3390/cimb46120826

**Published:** 2024-12-05

**Authors:** Anna Kot, Dominika Koszewska, Błażej Ochman, Elżbieta Świętochowska

**Affiliations:** Department of Medical and Molecular Biology, Faculty of Medical Sciences in Zabrze, Medical University of Silesia, 19 Jordana, 41-800 Zabrze, Poland; s85876@365.sum.edu.pl (A.K.); s85874@365.sum.edu.pl (D.K.); d201228@365.sum.edu.pl (B.O.)

**Keywords:** MINK1, STRIPAK, striatins, TNIK, MAP4Ks, Wnt, Hippo, ROS, autoimmunity, cancer

## Abstract

Misshapen/NIKs-related kinase (MINK) 1 belongs to the mammalian germinal center kinase (GCK) family. It contains the N-terminal, conserved kinase domain, a coiled-coil region, a proline-rich region, and a GCK, C-terminal domain with the Citron-NIK-Homology (CNH) domain. The kinase is an essential component of cellular signaling pathways, which include Wnt signaling, JNK signaling, pathways engaging Ras proteins, the Hippo pathway, and STRIPAK complexes. It thus contributes to regulating the cell cycle, apoptosis, cytoskeleton organization, cell migration, embryogenesis, or tissue homeostasis. MINK1 plays an important role in immunological responses, inhibiting Th17 and Th1 cell differentiation and regulating NLRP3 inflammasome function. It may be considered a link between ROS and the immunological system, and a potential antiviral target for human enteroviruses. The kinase has been implicated in the pathogenesis of sepsis, rheumatoid arthritis, asthma, SLE, and more. It is also involved in tumorigenesis and drug resistance in cancer. Silencing MINK1 reduces cancer cell migration, suggesting potential for new therapeutic approaches. Targeting MINK1 could be a promising treatment strategy for patients insensitive to current chemotherapies, and could improve their prognosis. Moreover, MINK1 plays an important role in the nervous system and the cardiovascular system development and function. The modulation of MINK1 activity could influence the course of neurodegenerative diseases, including Alzheimer’s disease. Further exploration of the activity of the kinase could also help in gaining more insight into factors involved in thrombosis or congenital heart disease. This review aims to summarize the current knowledge on MINK1, highlight its therapeutic and prognostic potential, and encourage more studies in this area.

## 1. Introduction

Misshapen/NIKs-related kinase (MINK) 1 is involved in the pathogenesis of numerous pathological conditions, such as autoimmune diseases, neurodegeneration, cardiovascular system malfunctions, and cancer [1,2,3,4]. It belongs to the family of mammalian germinal center kinase (GCK) and is also known as mitogen-activated protein kinase kinase kinase kinase 6 (MAP4K6), MINK, YSK2, B55, or ZC3 [5]. There are three known MINK1 ohnologues—paralogs originating from whole genome duplications [6], which are TRAF2 and NCK-interacting protein kinase (TNIK), mitogen-activated protein kinase kinase kinase kinase 4 (MAP4K4), and Nik-related protein kinase (NRK). MINK1 has only one Drosophila homolog—Misshapen—and serine/threonine-protein kinase mig-15 (MIG-15) is the C. elegans homolog of all four ohnologues mentioned. Along with TNIK, NIK, MIG-15, Misshapen, and NRK, MINK1 belongs to a branch separated from the group I and II GCK kinases, with different evolutionary roots than the two mentioned groups [7]. GCK kinases are part of the family of Ste20-related kinases, which also include the p21-activated kinases (PAKs). The kinases play a role in signaling pathways involving mitogen-activated protein kinases (MAPKs), regulate cytokinesis and cell growth [2,3,4,5,6,7,8], and are highly conserved from yeast to mammals [8]. Importantly, MINK1 is a crucial regulator of various cellular and tissue processes, particularly in response to diverse stress signals, as well as in the context of cell development and differentiation [3,9]. In addition, MINK1 expression has been demonstrated in multiple tissues and organs, including nervous tissue, kidneys, cancer tissues, and immune system cells, which will be discussed in detail in the following sections of the text. As demonstrated by Dan et al. via northern blotting, the MINK transcript is present mainly in the brain, but also in the kidney, spleen, and heart. Moreover, the kinase expression is enhanced during postnatal brain development in mice [7].

Many proteins have been found to interact with MINK1. For example, Qu and Xu et al. used yeast two-hybrid screening to identify proteins binding to its coiled-coil and proline-rich domains (a.a. 290–952) [10]. The results reveal that MINK1 interacts with factors responsible for cytoskeletal organization and regulating cellular structure (SPTBN2, SSH3BP1, 14-3-3beta, 14-3-3zeta, MAP1B, KAP-3, and PKP4 [11]), control of cell cycle and apoptosis (14-3-3beta, 14-3-3zeta, and MAP1B [12]), signal transduction (SSH3BP1, Calmodulin III [13]), cell adhesion (CASK [14]), as well as cell polarity (CDC42) [10]. It has also been reported to interact with Rap2, which takes part in synaptic transmission [15], and with TNIK, which plays a role in Wnt signaling [16]. Furthermore, there are many studies available that address the issue of the expression and function of MINK1 in various physiological and pathological conditions. MINK1, through its interactions with appropriate signaling pathways and biological processes, may be involved in the pathogenesis and progression of many diseases, including cancer, diseases of the nervous system, cardiovascular system, and immune system disorders among others. Detection of the expression level of MINK1 in human tissues could be a potentially useful prognostic marker in many pathological states, such as breast cancer, head and neck squamous cell carcinoma (HNSC), hepatocellular carcinoma (HCC), or Alzheimer’s disease. In addition, modulating MINK1 activity in autoimmunity, neurodegenerative diseases, or malignancies could improve the clinical outcomes of patients [1,3,17,18,19]. Understanding the kinase function in depth may give important insights into the exact biological processes behind them, allowing for developing new target therapy possibilities or prevention strategies in the future. The aim of this review is to highlight the clinical potential of MINK1, summarize the most important data on the role and interactions of MINK1 in cellular processes and relevant signaling pathways, and encourage further studies on the topic. To our knowledge, this is the first review attempting to summarize the current knowledge on MINK1.

## 2. MINK1 Structure and Cellular Localization

The human MINK gene is located on chromosome 17. The MINK transcript consists of 4848 base pairs and encodes a polypeptide of 1300 amino acids [20]. Hu et al. found that human MINK exists in multiple spliced versions. The authors obtained five alternative spliced isoforms of human MINK with different expression patterns. They described the isoform MINK1 (hMINK-delta) as containing 1295 amino acids, lacking 37 amino acids from amino acids 696 to 732, compared to the longest alternative spliced human isoform, hMINK-alpha. In this study (in contrast to the previous one [7]), human MINK1 was found to be predominantly expressed in skeletal muscles [20].

MINK1 can be dissected into four main domains [10]: the N-terminal, conserved kinase domain, a coiled-coil region (a.a. 394–495), a proline-rich region, and a GCK, C-terminal domain (a.a. 953–1295), up to 90% identical to the corresponding regions of other NIK-related kinases [7]. The C-terminal end of the kinase contains as well the Citron-NIK-Homology (CNH) domain [16]. The coiled coil region was found to match the residues 348–442 of the exocyst complex component SEC3, which regulates actin indirectly [21]. The GCK domain was shown to contain WD-40 motifs [10]. Qu et al. suggested that this domain may be responsible for substrate binding because beta-propeller folds in WD-motifs take part in protein–protein interactions [22]. Indeed, this domain was responsible for interactions with Rap2 in neurons [23] or the kinase domain inhibition in the Wnt non-canonical signaling [16]. Based on the complexity of the proline-rich domains of TNIK and MINK, and less abundant corresponding regions of NIK, it is probable that MINK emerged from TNIK, and TNIK evolved from NIK in the process of gene duplication and fusion [10].

**Figure 1:**
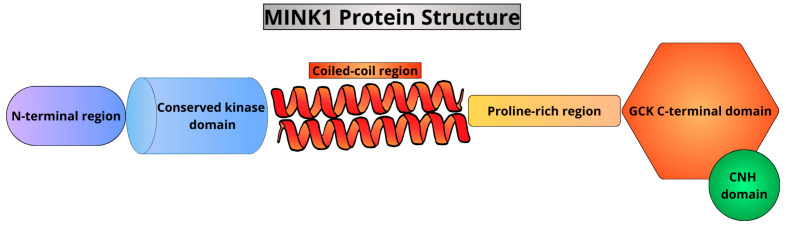
Schematic representation of the MINK1 protein structure, highlighting its major domains. The protein consists of an N-terminal domain (purple), a conserved kinase domain (blue) responsible for enzymatic activity, a coiled-coil region (residues 394–495; red) involved in actin regulation, a proline-rich region (yellow), and a GCK C-terminal domain (residues 953–1295; orange), containing WD-40 motifs crucial for substrate binding and protein–protein interactions. The GCK domain also includes a Citron-NIK-Homology (CNH) domain (green), implicated in additional regulatory functions.

The kinase is predominantly present in cytosol. It also localizes at tips of protrusions and vesicular structures, as well as in plasma membranes in the areas of contact of two neighboring cells. On the contrary, it has not been detected in the plasma membrane with no contact with other cells [15,24]. The schematic structure of the MINK1 protein is demonstrated in Figure 1. MINK1 localization in cells is dependent on various factors, and the distribution of its cleavage products may be different depending on their structure [16]. Treatment with specific antibodies revealed that MINK is cleaved in vivo into shorter N-terminal fragments, containing different lengths of central domain, and short C-terminal, inhibitory fragments. Cleavage sites within the central domain likely determine the subcellular localization of the polypeptides. Thus, polypeptides containing a longer central domain and kinase domain are directed to the cytoplasm. Consequently, the kinase and C-terminal domain are situated mainly in the cell nucleus, although the C-terminal domain may be located as well in the cytoplasmatic membrane. The N-terminal region can be found in fibrillar cytoplasmic structures of some cells, while the central domain is localized predominantly in the plasma and the nuclear membrane [16]. Surprisingly, the main endogenous MINK polypeptide seems to be an N-terminal polypeptide with kinase activity but without the CNH domain. On the other hand, little full-length kinases exist in embryos in vivo.

## 3. Embryogenesis and Cellular Signaling Pathways

### 3.1. Wnt Signaling

#### 3.1.1. MINK1 in Planar Cell Polarity and Convergent Extension

MINK1 has been implicated in the Wnt non-canonical signaling [25] and the role of MINK1 in planar cell polarity and convergent extension has been studied by Daulat et al. [9]. Tissue polarity, or planar cell polarity (PCP) is a form of polarity observed in vivo in complex structures, for example, in some epithelial cells polarized orthogonally to the apicobasal axis. Its manifestations can be found in the orientation of wing hair in drosophila, as well as in the organization of stereocilia in the inner ear [26,27,28]. PCP establishment and maintenance requires numerous proteins, including Flamingo (Fmi), Dishevelled (Dsh), Van Gogh (Vangl), Frizzled (Fz), Diego, and Prickle (Pk) [29,30,31,32,33]. Similar mechanisms regulate convergent extension (CE)—elongation and narrowing of a field of cells—during gastrulation in vertebrates, and both processes involve the Wnt non-canonical pathway [34,35]. Outside this function, the Wnt non-canonical signaling plays a role in the maintenance of stem cells and regulation of directional cell movements, including cancer cell metastasis [35,36,37,38,39].

Using mass spectrometry and Western blotting, Daulat and colleagues [9] showed that the kinase is associated with Prickle in HEK293T cells. They found that MINK1 phosphorylates Prickle on a conserved threonine residue at position 370 is critical for its localization within plasma membrane puncta in complexes with Vangl protein. It also took part in the regulation of Pk endosomal trafficking dependent on Rab5 during the establishment of CE in Xenopus embryos. Both Pk and Vangl had been shown to be required for their punctum localization in the cell membrane [40], but this study pointed out the importance of MINK1 in the process. Mikryukov and Moss [16], on the other hand, examined the interactions between TNIK and MINK in the process. Both kinases were found to play an essential role in the non-canonical PCP pathway in Xenopus embryos and an antagonistic role in the canonical ß-catenin-dependent signaling. This finding is consistent with previous discoveries that the Wnt non-canonical pathway can inhibit the Wnt β-catenin-dependent signaling [35,36,37,38,39]. The xTNIK and xMINK cDNAs examined in Xenopus encoded proteins in close relation to their orthologs in humans. The kinases were interchangeable for CE and suppressed the canonical Wnt signaling when activated simultaneously. The knockdown of either kinase resulted in a shortening of the anterior–posterior (A/P) axis depending on the dose, led to a retardation of the onset of gastrulation and the closure of the blastopore, as well as delayed migration of the dorsal or ventral blastopore lip. Mikryukov and Moss suggested that xMINK may act downstream from Dsh in PCP. While TNIK was found to be required for ß-catenin-dependent Wnt signaling [41,42], MINK was not necessary for canonical signaling but strongly opposed to TNIK catalytic activity. The full-length kinases seemed to co-localize in the cytoplasm of animal cap blastomeres, to form homo- and hetero-dimers, and to obtain autoinhibited via interactions of their CNH domains with the kinase domains. This inhibition may be the reason why the simultaneous expression of both full-length kinases inhibits Wnt canonical signaling.

#### 3.1.2. Interactions with Other Protein Complexes and the Implications for Cell Motility

Further studies were aimed at examining the mechanisms of the PCP/Wnt signaling in cancer cell dissemination [43]. PRICKLE1 knockdown in basal breast cancer cells led to decreased cell migration and proliferation, the formation of thick actin bundles, and the increased activity of ß1-integrin at the cellular surface. Similar results were achieved upon MINK1 downregulation. Staining with an anti-VINCULIN antibody revealed larger fatty acids (FAs) localizing at the tips of actin bundles. Notably, the decrease in cell motility could not be rescued by the expression of PRICKLE1 resistant to MINK1 phosphorylation or PRICKLE1 unable to interact with MINK1, which means that both PRICKLE1-MINK1 interaction and PRICKLE1 phosphorylation by MINK1 are essential for cell motility.

The authors also showed that a member of the mammalian target of rapamycin (mTOR) C2 complex, RICTOR, can bind to PRICKLE1 and that PRICKLE1-MINK1-RICTOR complex integrity is essential for AKT (protein kinase B, PKB) activation, focal adhesion regulation, as well as migration of cancer cells. The authors demonstrated that malfunctions in these interactions were associated with disrupted cell dissemination in breast cancer in xenograft assays. PRICKLE1 upregulation was related to poor metastasis-free survival in basal breast cancers. At the same time, PRICKLE1, MINK1, or RICTOR downregulation resulted in altered FA dynamics, integrin internalization, and polarization defect, as well as a decrease in cell migration. The authors identified RICTOR as a PRICKLE1 interactor and showed that MINK1 is essential in the formation of the PRICKLE1-RICTOR complex, highlighting the importance of the PRICKLE1-MINK1-mTORC2 complex in AKT phosphorylation. They suggested that MINK1 controls PRICKLE1-mTORC2 localization in migrating cells at their leading edge, which is necessary for AKT phosphorylation by mTORC2.

LL5β (PHLDB2) was identified as a direct target of the MINK1-Prickle1 complex [44]. Notably, MINK1 was essential for the binding of LL5β to Pk, as deletion of the LIM domain required for MINK1 association to PRICKLE1 led to the abolishment of PRICKLE1-LL5β association. MINK1 could phosphorylate LL5β in two residues: threonine 894, which is conserved among vertebrates and which is located within the domain required for cytoplasmic linker associated protein (CLASP) binding [45], and threonine 217. Additionally, the MINK1 inhibition or silencing increased the size of focal adhesions and cell spreading in MDA-MB-231 cells. Targeting MINK1 paralogs, MAP4K4, and TNIK did not lead to similar results. The loss of MINK1 also caused significant PRICKLE1 relocalization, from the actin-rich regions of the plasma membrane to actin bundles, and the mutation of LL5β at serine 892 and threonine 894 had the same effect. These results show that MINK1 stabilizes PRICKLE1 localization at the plasma membrane and promotes its association with LL5β. Moreover, the interaction between LL5β and CLASP2, a protein of which association with LL5β takes part in the anchoring of microtubules to the plasma membrane [45,46], seemed to be dependent on the LL5β phosphorylation by MINK1. This phosphorylation event was also required for cell motility in triple-negative breast cancer (TNBC).

**Figure 2:**
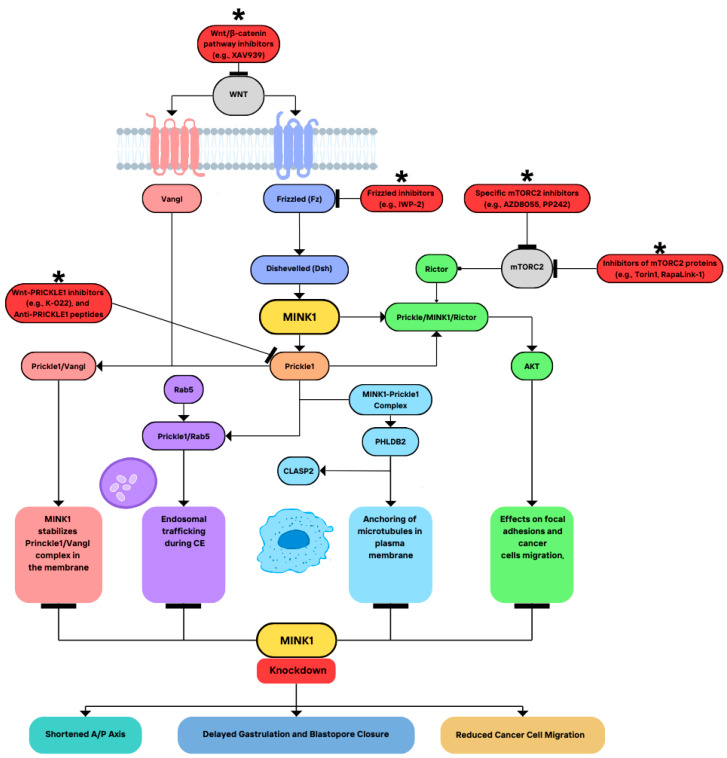
The signaling interactions between the Wnt/Frizzled pathway, MINK1, Prickle1, and their downstream effects on cellular processes. Fz—Frizzled; Dsh—Dishevelled; MINK1—Misshapen-like kinase 1; mTORC2—mammalian target of rapamycin complex 2; AKT—protein Kinase B; Vangl—Van Gogh-like protein; Prickle1—Prickle-like protein 1; Rab5—Ras-related protein Rab-5; CE—convergent extension; CLASP2—cytoplasmic linker associated protein 2; PHLDB2—pleckstrin homology-like domain Family B Member 2; and A/P axis—anterior–posterior axis, *—the possible inhibitors of the relevant components in the presented signaling pathways.

The role of MINK1 in Wnt non-canonical signaling has important clinical implications. As the Wnt signaling plays a significant role in the onset and progression of cancer [39,47,48], further research on the topic may help shed some light on the role of MINK1 in tumorigenesis. For example, MINK1 knockdown results in decreased breast cancer cells’ ability to invade secondary organs in vivo in comparison to control cells. It has been suggested that targeting the AKT-mTOR pathway and the disruption of PRICKLE1-RICTOR interaction could be a potential option for basal breast cancer treatment [43]. How MINK1 activity is influenced by extracellular signals or Wnt/PCP-related receptors remains unclear and should be addressed in other studies. Moreover, its interactions with other Pk-related proteins, such as ARHGAP21 and ARHGAP23, should be examined. In the future, MINK1 could serve as a therapeutic target in TNBC and other cancers [44]. To summarize, MINK1 is an important player in Wnt non-canonical signaling, with roles in PCP, CE, and cancer cell motility. Its interaction with PRICKLE1 and involvement in mTORC2-AKT signaling highlights its importance in cell migration and tumorigenesis. The illustration of the interactions of MINK1 with the WNT signaling pathway, along with the relevant cellular effects, is presented in Figure 2. The role of MINK1 in tumorigenesis will be discussed later in more detail.

### 3.2. RAS Proteins

MINK has been implicated in the growth arrest induced by the Ras pathway in human ovarian surface epithelial (HOSE) cells [4]. Rat sarcoma (RAS) proteins control the proliferation, apoptosis, migration, and survival of cells. They belong to the GTP-ases family and are represented by the products of three RAS genes. The most important isoforms are RAS4A, KRAS4B, NRAS, and HRAS, which all consist of conserved G domains responsible for signal transduction and C-terminal hypervariable regions (HVRs), allowing for RAS binding to membranes. The last four C-terminal amino acids undergo posttranslational changes, such as proteolysis, iso-prenylation, and methylation, thus modifying RAS localization [49,50]. RAS-GTP acts through two main pathways, rapidly accelerated fibrosarcoma (RAS–RAF)– mitogen-activated protein kinase kinase (MEK)–extracellular signal-regulated kinase (ERK) and Phosphoinositide 3-kinase (RAS–PI3K)–AKT–mTORC, to regulate cell behavior [51,52,53,54]. Mutations in RAS have serious oncological implications and are often a sign of poor prognosis for tumor patients [55,56,57]. Mutant RAS can be found in approximately 19% of cancer cases [58].

Nicke and colleagues found that oncogenic RAS caused a senescent-like phenotype in the majority of the cells. MINK was identified as one of three genes of which products could be involved in Ras-induced growth arrest [4]. Ras induction increased significantly MINK mRNA and protein levels. MEK inhibitor UO126 could abolish the activation of MINK by Ras, suggesting that the Raf/MEK/ERK pathway mediates this activation. MINK knockdown abolished p38 MAPK induction, partially inhibited Ras-induced p21^WAF1/CIP1^ elevation, and decreased cyclin A levels. Notably, in hs68 fibroblasts, Raf1 induction also led to decreased cell proliferation and increased p38 activation, which could be reversed by MINK knockdown. Nicke and colleagues suggested that MAP3K5 (Ask1) may serve as a candidate to connect p38 MAPK activation and MINK. In the study, the addition of N-acetyl cysteine (NAC) resulted in the lowered activity of p38 MAPK and MINK, while H_2_O_2_ addition increased their phosphorylation and activity rapidly, implying that Ras-induced reactive oxygen species (ROS) positively regulate the activation of both MINK and p38 MAPK. In conclusion, Ras induced MINK expression, which likely acted downstream from the Raf/MEK/ERK pathway, and was activated in a ROS-dependent manner. MINK controlled p38 MAPK, and thus p21^WAF1/CIP1^ induction, leading to the growth arrest. The authors found no evidence that MINK participates in Ras-dependent transformation and the maintenance of the transformed phenotypes in human tumor cells [4].

**Figure 3:**
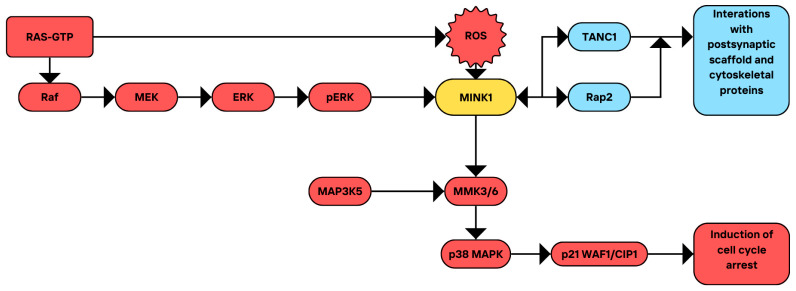
MINK1 and RAS signaling interactions. ERK—extracellular signal-regulated kinase; MEK—mitogen-activated protein kinase kinase; RAS-GTP—Ras guanosine triphosphate; Raf—rapidly accelerated fibrosarcoma kinase; pERK—phosphorylated extracellular signal-regulated kinase; MINK1—Misshapen-like kinase 1; ROS—reactive oxygen species; MAP3K5—mitogen-activated protein kinase kinase kinase 5; MMK3/6—mitogen-activated protein kinase kinase 3/6; p38 MAPK—p38 mitogen-activated protein kinase; p21 WAF1/CIP1—p21 wild-type p53-activated fragment 1/cyclin-dependent kinase-interacting protein 1; Rap2—Ras-related protein Rap2; and TANC1—tetratricopeptide repeat, ankyrin repeat, and coiled-coil containing 1.

Later studies showed that MINK interacts with wild-type and a constitutively active Rap2 (a Ras-like G protein [59]) mutant, but not with Rap1 nor Ras [15]. When coexpressed, MINK and Rap2 significantly colocalized in vesicular structures. Moreover, MINK interacted with tetratricopeptide repeat, ankyrin repeat, and coiled-coil containing 1 (TANC1)—a postsynaptic density protein [60]—and induced its phosphorylation under the control of Rap2, which would be an interesting target for further research. The summary of current knowledge on the interactions of MINK1 with the RAS signaling pathway, along with the relevant cellular processes and implications discussed here, is presented in Figure 3.

### 3.3. JNK Signaling

MINK1 also takes part in pathways that involve the JNK signaling [3,7]. The c-Jun N-terminal kinase family (JNK) belongs to the family of mitogen-activated protein (MAP) kinases and consists of 10 splice versions of products of three genes: JNK1 (MAPK8), JNK2 (MAPK9), and JNK3 (MAPK10) [61]. Among them, JNK3 is abundantly present in neurons, but can also be found in the heart and the testis, while JNK1 and JNK2 are distributed less specifically [62]. JNK1 and JNK2 play a role in immunological processes and nervous system development: they act to maintain physiological processes in the central nervous system (CNS) [62,63,64]. JNK3 controls brain function in the state of both physiology and pathology. It has been demonstrated to take part in the development of the brain, neurite formation, and plasticity, as well as in memory and learning [62,65,66]. In pathological conditions, JNK3 has been shown to transduce neural degeneration signals and to be responsible for JNK hyperactivity in detrimental stress in the adult brain, in the event of hypoxia, ischemia, or epilepsy [66,67,68]. Additionally, JNK signaling has been implicated in cytokine production and secretion of natural killer (NK) cells, oncological and drug-resistance models, or myeloproliferative diseases [69,70,71,72].

#### MINK1 Interactions with the JNK Family

**Figure 4:**
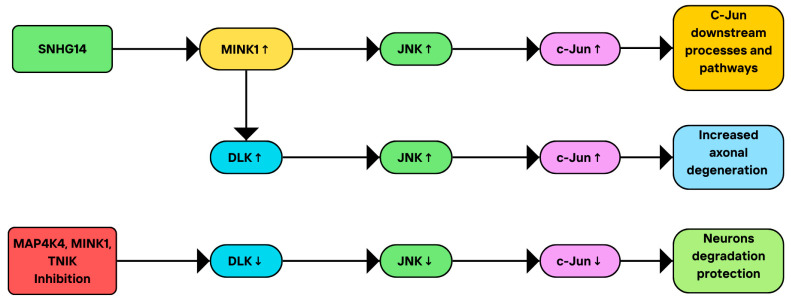
MINK1 and JNK signaling pathway interaction. MAP4K4—mitogen-activated protein kinase kinase kinase kinase 4; MINK1—Misshapen-like kinase 1; TNIK—Traf2- and Nck-interacting kinase; DLK—dual leucine zipper kinase; JNK—c-Jun N-terminal kinase; c-JUN—c-Jun proto-oncogene; and SNHG14—small nucleolar RNA host gene 14.

Drosophila Ste20-related kinase misshapen (Msn) has been shown to act upstream from DJNK [73] and several studies demonstrated the importance of MINK1 in pathways involving JNK signaling. MINK can activate JNK1, as reported by Dan and colleagues [7]. In addition, the human MINK kinase domain has been shown to increase JNK activity, and its C-terminal domain presented similar effects, but to a lesser extent. Variable regions of MINK on the other hand did not affect JNK activation [74]. Another study highlighted the role of MINK in neurodegeneration. Dual leucine zipper kinase (DLK) is essential for axonal degeneration due to growth factor deprivation by activating JNK phosphorylation and transmitting the stress signal to the nucleus [75]. Larhammar et al. showed that the Ste20 kinases MAP4K4, MINK1, and TNIK regulate DLK/JNK signaling in neurons and that their simultaneous inhibition decreases DLK activation and c-Jun phosphorylation, protecting neurons from degeneration [3]. It has also been demonstrated that small nucleolar RNA host gene 14 (SNHG14) may upregulate MINK1, leading to increased c-Jun phosphorylation in THP-1 human macrophage-like cells [76]. The summary of the presented studies on the role of MINK1 in the JNK signaling pathway, including its role in JNK activation and cellular consequences, is illustrated in Figure 4.

The reports mentioned indicate that MINK1 acts upstream of JNK to promote its activation. This fact may have important clinical implications, which will be discussed later in the text.

### 3.4. The Hippo Pathway

The Hippo pathway, conserved from Drosophila to mammals, is considered a key regulator of cell fate during embryogenesis, tissue homeostasis, and organ size [77,78,79,80,81]. Its core components in Drosophila are Ste20-like kinase Hippo (Hpo), Salvador (Sav)—its adaptor protein, Warts (Wts)—the NDR family kinase—with its adaptor Mats, and Yokie (Yki), the transcriptional effector [82,83,84,85,86]. In mammals, the basic Hippo proteins are Mammalian Ste20-like kinases 1/2 (MST1/2, Hpo homologs) with the adaptor protein Sav family WW domain-containing protein 1 (SAV1, Sav homolog), large tumor suppressor 1/2 (LATS1/2, Wts homologs), MOB1A/1B (Mats homologs)—their adaptor proteins, as well as the Yes-associated protein (YAP), and the transcriptional co-activator with PDZ-binding motif (TAZ), the two Yki homologs. The kinase cascade may be regulated by several factors, including apical-basal polarity, cell–cell contact, serum deprivation or energy stress, and extracellular hormones [87,88,89,90,91,92,93,94]. Deleting or overexpressing certain elements of the Hippo pathway in mice models led to tumorigenesis and organ enlargement, and mutations in genes encoding the proteins of the Hippo signaling are implicated in numerous human malignancies [84,95,96].

Meng et al. showed that MAP4Ks are regulators of LATS1/2 and YAP/TAZ and that LATS1 is a potential substrate for MINK1 and other MAP4Ks, demonstrating that MAP4Ks are important components of the Hippo pathway [97]. The study revealed that LATS is essential for the regulation of YAP/TAZ, while MST is not, as its depletion did not abolish YAP phosphorylation in response to various signals. Additionally, MAP4Ks, including MINK1, are likely to directly phosphorylate and activate LATS independently from MST. MAP4K4, closely related to MINK1, could further induce YAP phosphorylation. Importantly, due to functional redundancy, deletion of MAP4K4 alone does not decrease YAP phosphorylation significantly, while this effect is achieved via additional deletion of MAP4K6 (MINK1) and MAP4K7. Still, MAP4K4/6/7 deletion does not abolish YAP phosphorylation completely, suggesting that MAP4Ks exhibit parallel and redundant effects on YAP phosphorylation and inactivation.

**Figure 5:**
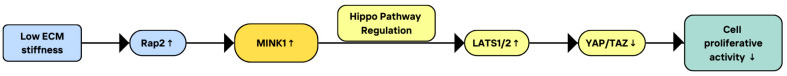
MINK1 and Hippo pathway regulation. ECM—extracellular matrix; Rap2—Ras-related protein 2; MINK1—Misshapen-like kinase 1; LATS1/2—large tumor suppressor kinase 1/2; and YAP/TAZ—Yes-associated protein/WW domain-containing transcription regulator 1.

The previously mentioned Ras component, Rap2, has also been implicated in the Hippo pathway and its regulation in response to extracellular signals [98]. The GTP-ase is activated in the conditions of low extracellular matrix (ECM) stiffness and stimulates MAP4Ks, including MINK1, as well as Rho GTPase activating protein 29 (ARHGAP29). This leads to the activation of LATS1/2 and subsequent YAP/TAZ inhibition. These findings suggest that MINK1 plays a role in mechanotransduction as a part of the Hippo signaling. Moreover, as MINK1 directly interacts with Rap2 [15], it would be interesting to further study the Rap2-MINK1 interactions in mechanosignaling and the Hippo pathway. The main findings and interactions of MINK1 with the Hippo pathway signaling, along with the proteins it regulates, are illustrated in Figure 5.

The role of MINK1 in the mentioned signaling pathways suggests its special place in the regulation of various physiological processes regulating cell function, embryogenesis, and tissue homeostasis, which has also implications for organ function, as well as the function of whole bodily systems. There is still a lot to uncover when it comes to the role of MINK1 in the Wnt, Ras, JNK, and Hippo signaling. Its interactions with other elements of the pathways could be studied in the future. Known pathological conditions involving MINK1 and these signaling pathways will be discussed later in the text.

### 3.5. MINK1 in the STRIPAK Complexes

Importantly, MINK1 has been suggested as a novel STRIPAK component [99]. Striatin (STRN), S/G2 nuclear autoantigen (SG2NA, STRN3), and zinedin (STRN4) together form the mammalian striatin family. These proteins are expressed predominantly in the nervous system and are likely important for its function, but they can be also detected in other tissues. They act as scaffolding proteins and can form large signaling complexes, which are then responsible for various essential cell functions [100,101]. Such complexes usually contain one of the two subunits (structural subunit A and catalytic subunit C) of protein phosphatase 2A (PP2A), an eukaryotic serine/threonine phosphatase [102]. A proteomic analysis uncovered various other components, including germinal center kinase III (GCKIII), interacting with the striatin family together with PP2A [103]. These complexes are now known as the striatin-interacting phosphatase and kinase (STRIPAK) complexes, and along with them, many STRIPAK-like complexes, containing PP2A and GCKIII, have been discovered. The STRIPAK complexes have been implicated in conditions such as heart disease [104,105,106], diabetes [107,108], autism [109,110], cancer [111,112], or cerebral cavernous malformation (CCM) [113,114,115]. Recent reports also highlight the prognostic role of STRIPAK components in human cancer [17].

**Figure 6:**
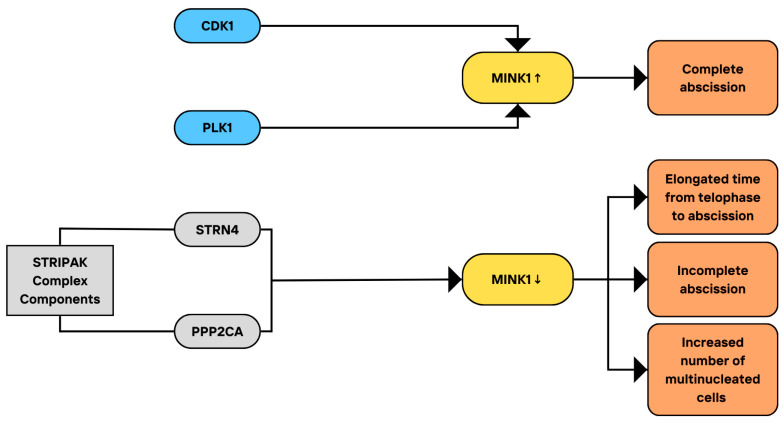
MINK1 and STRIPAK complex. STRIPAK—striatin-interacting phosphatase and kinase; STRN4—Striatin 4; PLK1—Polo-like kinase 1; CDK1—cyclin-dependent kinase 1; MINK1—Misshapen-like kinase 1; and PPP2CA—protein phosphatase 2 catalytic subunit A.

Both MINK1 and STRN4 (Zinedin) are necessary for abscission, which is the final stage of cytokinesis [99]. MINK1 silencing, performed via two siRNAs, resulted in an increased number of multinucleated cells, and similar effects were obtained by using catalytically inactive kinase. As was observed by using time-lapse imaging, MINK1 knockdown led to the inability to complete the abscission in 20% of the cells, and to the delayed abscission in most of them, with elongated time of the progression from telophase to the completion of abscission. MINK1 is also phosphorylated in mitosis, and this phosphorylation may be induced by CDK1 and Polo-like kinase 1 (PLK1), the kinases important for mitosis [116,117,118], but it is not certain whether these kinases can phosphorylate MINK1 in vivo. Moreover, Zinedin (STRN4) and protein phosphatase 2A catalytic subunit, alpha isoform (PPP2CA), immunoprecipitated with MINK1, and FAM40A/B (STRIP1/2), sarcolemma-associated protein (SLMAP), and cortactin-binding protein 2 N-terminal-like (CTTNBP2NL) formed complexes with it. These proteins have been recently recognized as STRIPAK components [103]. STRN4 was also necessary for the completion of the cytokinesis, and similarly to MINK1, STRN4 depletion led to a decreased number of cells that could complete the abscission. STRN4 was not sufficient to reduce MINK1 activity, but with the protein phosphatase 2 scaffold subunit alpha (PPP2R1A)/PPP2CA complex, it led to MINK1 inhibition. This inhibitory mechanism appears to be similar to the negative regulation of GCKIII kinases by striatin [119]. Notably, PP2A is known to be inhibited in mitosis and becomes reactivated at the mitotic exit [120,121,122], which corresponds to the increased phosphorylation of MINK1 in mitosis and its dephosphorylation within an hour after releasing the cells from nocodazole-induced cell cycle arrest [99]. Together, these results indicate that MINK1 is essential for the completion of cell division by regulating the abscission and that its activity is dependent on the mentioned STRIPAK components, which can also play a role in mitosis. MINK1 is thus a STRIPAK interactor. However, it is unknown whether the MINK1 regulation by STRN4 is meaningful for mitosis in vivo, and further research on the topic would be needed. It is also not clear if STRN4 regulates cytokinesis through interactions with STRIPAK. The key associations and interactions of MINK1 with the STRIPAK complex, including factors influencing MINK activity and cellular effects related to it, are presented in Figure 6.

Many questions regarding STRIPAK, its functions, and its regulation, remain unanswered. It is also not clear what functions independent from STRIPAK are performed by its components. The exact role of MINK1 as the STRPAK interactor remains unknown as well, and hopefully more light will be shed on the issue in the future.

## 4. The Role of MINK1 in Immunological Processes and Diseases

The role of MINK in numerous inflammatory diseases has been evaluated extensively, and the results clearly show its essential role in the function of the immune system. MINK1 has been implicated in conditions including rheumatoid arthritis, asthma, and SLE [1,19,123], and its activity regulates the immune responses on many levels, as discussed below.

### 4.1. Reactive Oxygen Species, Inflammation, and the Immune System

Reactive oxygen species (ROS), which can be produced by cells involved in the host-defense responses, are essential signaling molecules and inflammation mediators but are also known for promoting tissue injury and taking part in various immunological disorders [124]. ROS seem to boost the expression of MINK1. Nicke et al. showed that MINK may be involved in the growth arrest induced by the Ras pathway in human ovarian surface epithelial (HOSE) cells [4]. They demonstrated as well that MINK activation is markedly decreased after the addition of the antioxidant NAC and pyrrolidine dithiocarbamate plus ascorbate. Consistently, a major elevation of MINK level was observed upon the addition of H_2_O_2_ [4]. Another study revealed that the removal of ROS decreases MINK1 kinase activity in vitro [125]. A similar effect occurs in primary mouse CD4^+^ T cells [126]. However, further research is needed to elucidate the exact mechanism in which ROS boost MINK1 activity.

MINK1 has been reported to influence many key components of the immune system, including Th17 cells [126] that play an important role in autoimmune diseases [127,128,129]. Th17 cells play a crucial role in inducing and regulating inflammatory states [127,130]. They are significantly more encephalogenic than Th1 cells and induced effectively severe experimental autoimmune encephalomyelitis (EAE) in mouse models [127]. Th17 cells have also been reported to take part in the pathogenesis of asthma [131] and systemic lupus erythematosus (SLE) [132] and likely play a role in rheumatoid arthritis [133].

Fu et al. showed that MINK1 has a suppressive effect on Th17 cell differentiation [126]. The authors found that in the mouse experimental autoimmune encephalomyelitis (EAE) model, MINK1^−/−^ mice showed a 2–3 times increased number of Th17 and Th1 cells compared to WT mice upon stimulation with PMA and ionomycin. There was also a marked increase in the expression of Th17 lineage-specific genes and Th1 signature Tbx21 in MINK1-deficient cells, thus showing that cell differentiation towards Th17 and Th1 cells is likely favored in the absence of MINK1. The studies of the CD4^+^CD44^+^ T cell population also revealed a major increase (3–4 times) in the number of Th17 MINK1-deficient cells compared to WT cells. The number of Thγδ cells producing IL-17A in MINK1^−/−^ mice was elevated as well. The effect on Th17 cell differentiation occurred in vivo through the T-cell intrinsic mechanism. MINK1^−/−^ mice also displayed enhanced EAE severity with more prominent demyelination in the central nervous system and the spinal cord, accompanied by increased IL-17A production by CD4^+^ T cells. The authors demonstrated that the negative regulation of Th17 cell differentiation in MINK1 deficiency may occur through SMAD2 inhibition by direct phosphorylation of its T324 residue. Interactions between MINK and SMAD have been studied as well by Kaneko et al., who demonstrated that SMAD can be inhibited by phosphorylation in alpha-helix 1 by Misshapen in Drosophila and by the mammalian orthologs TNIK, MINK1, and MAP4K4 [134]. Notably, Daulat et al. in their study did not find SMAD2 among proteins phosphorylated by MINK1 [44]. This indicates that MINK1 substrates may depend on cell context.

Interestingly, NAC increased the number of Th17 cells in the mouse EAE model, enhancing the severity of the disease. The elevation of the Th17 cell level also occurred in WT mice receiving NAC in drinking water, while the effect did not take place in MINK1^−/−^ mice. Although NAC supplementation did not aggravate symptoms of EAE in mice in vivo, which may be due to its effect on other cells, these results suggest that MINK1 may be the long-searched link between ROS and reduced differentiation of Th17 cells [126]. Other studies on the effect of ROS on Th17 cell development present contradictory results. Zhi et al. showed that the addition of NAC suppresses Th17 cell differentiation [133]. They suggested that in the absence of IEX-1, ROS formation in mitochondria may be the factor responsible for the increased number of Th17 cells. In another study, NAC treatment was demonstrated to affect positively Th17 cell differentiation and the authors suggested that Th17 cell generation is mediated by ROS [135]. The research on MINK1 may explain in more depth how ROS and Th17 cell development are connected.

MINK1 influences as well the function of the nucleotide-binding domain, leucine-rich-repeat-containing family, pyrin domain-containing 3 (NLRP3/NALP3) inflammasome [125]. NLRP3 proteins are expressed abundantly in neutrophils and macrophages, and the increase in their levels may be caused by stimulation with LPS in mouse models [136]. NLPR3 expression can be detected mainly in stratified non-keratinizing squamous epithelium. NLRP3 triggers an immune response by activating molecules such as IL-1β [137]. It has been shown to play a role in insulin resistance, type 2 diabetes (T2D), gout, and sepsis [138,139,140].

MINK1 is essential for NLRP3 inflammasome activation. In MINK1-deficient mice, the activation of NLRP3 inflammasome in bone marrow-derived macrophages (BMDMs) is impaired upon treatment with ATP, nigericin, and alum. The impairment resulted in lowered levels of L-1β cleavage, reduced maturation of caspase-1, as well as a decrease in IL-1β and IL-18 secretion [125]. However, the role of MINK1 has not been demonstrated for other inflammasomes, such as AIM2 or NLRC4. As MINK deficiency does not affect the expression of NLRP3, procaspase-1, and pro-IL-1β, it likely regulates the function of NLRP3 through protein phosphorylation. Consistently, overexpression of the MINK1 kinase domain rescues the secretion of IL-1β induced by ATP, as well as caspase-1 activity. Immunoprecipitation and immunofluorescence revealed that MINK1 may interact directly with NLRP3, but not with other inflammasome components. Zhu et al. identified Ser725 as the residue of phosphorylation, which was essential for NLRP3 inflammasome activation. S725 phosphorylation also allowed for NLRP3 self-association.

The findings regarding MINK1 and NLRP3 inflammasome activation have significant clinical implications. MINK1 knockdown has been reported to boost the survival rate and decrease the disease severity in LPS-induced sepsis and alum-induced peritonitis in mouse models, lowering the levels of IL-1β and IL-18 in serum of MINK1^−/−^ mice compared to wild-type mice [125]. The overall inflammatory responses seem to be stronger in the presence of MINK1. Consistently, the maturation and secretion of IL-1β and caspase-1 are greatly comprised by the NAC treatment, which is not observed in MINK1^−/−^ cells. Prophylactic NAC treatment seems to alleviate acute inflammatory responses effectively, but MINK1^−/−^ mice show little improvement. Importantly, a similar mechanism occurs in humans. In peripheral blood mononuclear cells (PBMCs) derived from healthy donors, NAC lowered IL-1β secretion. In the human THP-1 cell line with MINK1 knockdown ROS, scavengers do not reduce the activation of NLRP3 inflammasome, while the effect does take place in cells with functional MINK1. These results demonstrate that MINK1 may exert a therapeutic effect in inflammatory diseases related to NLRP3 in humans.

### 4.2. Autoimmune Diseases and Other Conditions

Interesting studies have been performed regarding the role of MINK1 in asthma [1]. Asthma is a chronic inflammatory condition of the airways with heterogeneous etiology [141,142]. There are two main asthma phenotypes: T2-high (Type 2) and non-T2-high [143,144]. The second one has been connected to the enhanced activity of Th1 or Th17 cells [145]. Higher levels of IL-17 were connected to increased severity of the disease in asthma patients [131,146]. Th17 asthma is driven mainly by Th17 cells. In the study conducted by Chan et al., bronchial epithelial cells (BECs) could induce immune responses acting as antigen-presenting cells (APCs) and initiate predominantly Th17-dominant asthma in mice exposed to both house dust mites (HDM) and lipopolysaccharide (LPS) and Th2-dominant asthma in the HDM-exposed group. In the Th17-dominant asthma mouse model, the animals showed higher baseline lung resistance (RL) and peribronchial inflammatory cell infiltration than the T2 asthma mice [1].

Histological analyses revealed a marked decrease in MINK1 staining and increased methyl-CpG-binding domain protein 2 (MBD2) staining in the lungs of the mice in the Th17-asthma group [1]. Consistently, the mRNA levels of MBD2 were elevated, while the levels of MINK1 mRNA were lowered, which suggests the involvement of MINK1 and MBD2 in the development of asthma phenotype and severity. While MINK1 does not seem to affect MBD2 expression, MINK1 expression is increased when the MBD2 gene is silenced and significantly reduced when MBD2 is overexpressed. Moreover, exposure to HDM and LPS enhances MBD2 binding to the promoter region of the MINK1 gene, showing that MBD2 is the factor silencing MINK1 in Th17-dominant asthma. The authors demonstrated as well that MINK1 silencing resulted in an increase in Th17 cell differentiation and IL-17 levels and that these parameters showed a decrease upon MINK1 overexpression. These findings suggest that stimulating MINK1 expression or targeting MBD2 could be a potential strategy in the treatment of Th-17 asthma. It would be interesting to investigate the interactions between MINK1 and MBD2 in other conditions involving Th17 cells as well, as MBD2 has also been shown to regulate the development of Th17 cells [147].

Another clinical condition in which the essential role may be played by MINK1 is rheumatoid arthritis (RA). RA is a chronic disease characterized by autoinflammatory reactions and inflammatory changes in joints [148,149]. Recently, the role of lncRNA small nucleolar RNA host gene 14 (SNHG14) in RA pathogenesis has been studied [76]. SNHG14 has been previously shown to promote inflammatory responses and contribute to neurological impairment by upregulating rho-associated coiled-coil-containing protein kinase 1 in cerebral ischemia, sponging microRNA (miR)-136–5p [150]. Zhang et al. demonstrated that SNHG14 expression is enhanced in primary CD14+ macrophages and PBMCs of patients with RA in comparison to the control group [76]. SNHG14 silencing negatively affects cell proliferation. The knockdown of SNHG14 also results in decreased levels of cytokines IL-6, IL-1β, and TNF-α in LPS-stimulated pTHP-1 macrophages, which means that SNHG14 plays an important role in proliferation and proinflammatory cytokine secretion. Additionally, the authors showed that SNHG14 may bind to miR-17-5p.

MiR-17-5p overexpression leads to downregulation of MINK1 expression and the MINK1 gene was detected to be a possible interactor of miR-17-5p, as revealed with the use of the starBase [76]. The level of MINK1 is also significantly enhanced in LPS-stimulated pTHP-1 macrophages, and its decrease may be observed upon SNHG14 depletion. These results indicate that miR-17-5p may target MINK1 and that SNHG14 may increase the expression of the kinase. Additionally, MINK1 overexpression reduces the effects of SNHG14 silencing on cell proliferation and cytokine expression. This suggests that SNHG14 performs the mentioned functions by MINK1 upregulation. The overexpression of MINK1 could also reverse the decrease in the levels of p-JNK, p-ERK, and p-p38 caused by the SNHG14 silencing, indicating that SNHG14—via MINK1 upregulation—may take part in JNK signaling pathway activation. Thus, targeting MINK1 or SNHG14/MINK1 axis could be explored in the context of RA treatment in the future.

MINK1 has been also detected to be connected to systemic lupus erythematosus (SLE) risk [123]. SLE is an autoinflammatory disease involving diverse organs of the body [151,152]. MINK1 promoter interacts with open chromatin regions (OCR) associated with the risk of SLE, and its expression is enhanced in human follicular helper T cells (TFH) [123]. Targeting MINK1 with a MAP3/4K antagonist caused a decrease in IL-21 secretion by TFH cells. These results suggest that MINK1 plays a role in TFH cell function and may serve as a therapeutic target in SLE.

### 4.3. Viral Infections

MINK is involved as well in the replication of human enterovirus 71 (EV71) [153]. EV71 infection may cause hand, foot, and mouth disease, which is especially concerning in Asia, where the highest case fatality is observed. The disease symptoms may vary from mild to severe, involving neurological complications [154,155].

Leong et al. studied the involvement of MINK in EV71 infection [153], and the treatment with siRNA that targeted MINK-reduced viral replication. Interestingly, Leong et al. demonstrated the importance of MINK in the replication process of other human enteroviruses: EV71 strain 41, coxsackievirus A6 (CA6), and echovirus 7, as MINK knockdown led to the decrease in infectious virus titers in the three cases. MINK silencing using siRNA was found to reduce viral RNA and protein expression in human rhabdomyosarcoma (RD) cells. The authors thus suggested that MINK is essential for viral protein synthesis. The MINK activation through phosphorylation likely took place in the early phase of viral replication, as virus binding did not trigger MINK phosphorylation in the study. Phosphorylation of p38 MAPK, which was activated by MINK [7], was enhanced in infected cells between 6 and 8 h after the infection, and dropped in the next hours. On the contrary, the cells in the control group and cells infected with UV-inactivated EV71 demonstrated constant, but lower p38 phosphorylation [153]. Additionally, phospho-p38 MAPK levels were reduced in the presence of MINK siRNA, confirming the role of MINK in p38 MAPK activation during the replication of EV71. P38 phosphorylation was also demonstrated to be necessary for EV71 replication in RD cells. The authors concluded that active viral replication is needed for p38 MAPK activation, which is phosphorylated downstream from MINK, allowing for further viral replication and viral protein synthesis.

MINK knockdown also boosted the levels of the heterogeneous nuclear ribonucleoprotein A1 (hnRNP A1) in the nucleus, decreasing hnRNP A1 signals in the cell cytoplasm. Similar results were obtained upon the inhibition of p38 MAPK. The authors thus suggested that hnRNP A1 may be a target in MINK/p38 MAPK signaling. hnRNP A1 is a mainly nuclear protein, but it can be transferred to the cell cytoplasm, acting as an internal ribosome entry sites (IRES) transacting factor (ITAF) and promoting EV71 IRES translation, which is required for the initiation of viral protein translation [156,157,158]. These results offer insight into the mechanism of EV71 translation regulation by MINK/p38 MAPK signaling. MINK is therefore a potential antiviral target affecting the viral replication at the stage of translation of EV71 proteins and it should be further explored in this context [153].

### 4.4. MINK1 in Immunity

**Figure 7:**
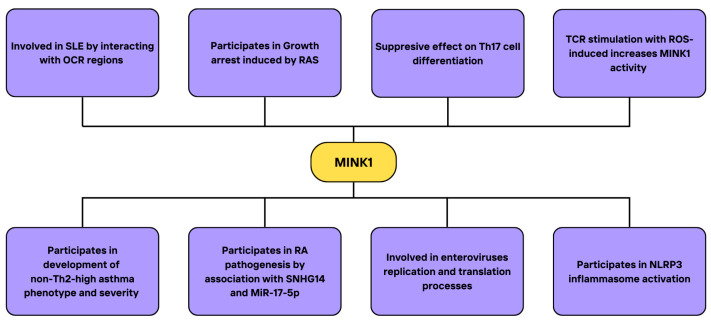
MINK1 in immunity. SLE—systemic lupus erythematosus; OCR—open chromatin regions; TCR—T-cell receptor; ROS—reactive oxygen species; NLRP3—NOD-like receptor family pyrin domain containing 3; MINK1—Misshapen-like kinase 1; RA—rheumatoid arthritis; and SNHG14—small nucleolar RNA host gene 14.

In conclusion, ROS enhance MINK1 expression, while ROS scavengers such as NAC reduce it. The kinase takes part in various immunological processes, suppressing Th17 and Th1 cell differentiation, regulating the secretion of cytokines and NLRP3 inflammasome activation, thus influencing the progression and severity of inflammatory states and autoimmunological diseases that involve the mentioned components, including sepsis, rheumatoid arthritis, asthma, SLE and others. The research on MINK1 offered a deeper insight into the pathogenesis of the mentioned conditions, explaining the possible role of ROS in their onset. Importantly, MINK was also reported to be necessary for the replication process of human enteroviruses, including EV71. Targeting MINK1 could serve as a potential therapeutic strategy for patients suffering from numerous inflammatory diseases, as well as viral infections. Inducing MINK1 expression in asthma or targeting the SNHG14/MINK1 axis in RA could be considered interesting directions for future research. At the same time, a careful examination of factors that influence MINK1 expression in autoimmune diseases could be beneficial in the context of developing new therapeutic approaches. A summary of the MINK1 interactions and its implications on cellular events, processes, and related diseases, along with molecules and signaling pathways regulated by MINK and MINK interactors, is demonstrated in Figure 7.

## 5. MINK1 in Cancer

MINK1 is a key component of various cellular signaling pathways implicated in cancer, including WNT/PCP, RAS, JNK, and Hippo signaling. It is also an interactor of STRIPAK complexes, which have been recently studied and reviewed in the context of tumorigenesis [17,159]. This indicates that MINK1 itself could be involved in the pathogenesis of cancer. Still, the role of MINK1 in malignancies remains blurry, as only a small number of studies tackling this issue are available.

### 5.1. Breast Cancer

Several researchers examined the function of MINK1 in breast cancer. The kinase could have a prognostic potential in neoplastic diseases, while its targeting could be useful in increasing patients’ sensitivity to therapies. Higher MINK1 levels were found to be correlated with shorter overall survival of the patients (*p* = 0.011) suffering from breast cancer [17]. Enhanced MINK1 expression was also associated with lower responses to drugs and chemotherapies, but the correlation was not statistically significant [17]. These results indicate that MINK1 could serve as a prognostic marker for patients with breast cancer and that its role in this context should be studied in other cancer types as well. Measuring its levels could be also beneficial for predicting patients’ responses to the treatment. However, it would be necessary to validate the results in independent studies, as Xinyu et al. utilized a historical collection of frozen tissues, which might have influenced the outcomes. In another study, Daulat et al. observed a strong decrease in the motility of breast cancer cells upon MINK1 downregulation [43]. As discussed earlier in the text, the reduction in PRICKLE1 and MINK1 levels led to decreased cell proliferation and the formation of thick actin bundles. The cells also exhibited a different phenotype with a larger nucleus diameter, increased cell surface area, and lack of lamellipodia in the event of MINK1 and PRICKLE1 depletion. In addition, MINK1, or RICTOR downregulation, resulted in altered FA dynamics, integrin internalization and polarization defect, and a decrease in cell migration. Daulat and colleagues demonstrated that the interaction between PRICKLE1 and MINK1, and PRICKLE1 phosphorylation in particular, is required for cell motility. It seems that cancer cell migration via disruption of FA dynamics is controlled by the PRICKLE1-MINK1-RICTOR complex. In conclusion, MINK1 is likely involved in the metastatic spread of cancer cells in breast cancer. Targeting MINK1 could thus be a potential therapeutic strategy for the disease. Another study showed that treatment with TNIK inhibitor KY05009 phenocopies the loss of MINK1 in triple-negative breast cancer (TNBC), and such downregulation resulted in increased focal adhesion size [44]. MINK1 regulated the interactions between CLASP2 and LL5β, which have been implicated in cell migration in other studies [160,161]. Consistently, the loss of MINK1 kinase activity decreased cell migration in the breast cancer cell lines [44]. Developing new MINK1 inhibitors should be a focus of future studies, as targeting MINK1 seems to be a promising strategy in TNBC treatment. However, the overall number of studies regarding MINK1 expression in human tumors is low; therefore, it is important to encourage more research on the subject.

### 5.2. Oral Squamous Cell Carcinoma

Interesting results were obtained regarding the role of MINK1 in oral squamous cell carcinoma (OSCC). In the study conducted by Mohanty et al., the authors uncovered that MINK1 function may be responsible for 5FU resistance in patients suffering from OSCC. It was also overexpressed in 5FU-resistant (5FUR) cells and in tumor tissues of non-responding patients [162]. The role of MINK1 expression in drug resistance has been implicated in another study as well [163]. The viability of chemo-resistant cells treated with 5FU was significantly decreased after MINK1 knockdown [162]. Moreover, MINK1 knockdown resulted in lowered tumor mass in response to 5FU treatment in mice, but no such effect was observed in the MINK1 WT group. Additionally, MINK1-depleted cells showed decreased migration and a lower number of thymidylate synthase and ABC transporters. At the same time, increased thymidylate synthase expression was found in sensitive OSCC lines when MINK1 was ectopically overexpressed. Additionally, MINK1-overexpressing cells were resistant to cell death induced by 5FU, but different results were obtained for kinase-dead MINK1 overexpression. Overall, MINK1 kinase activity seems to be a key factor mediating 5FU resistance in OSCC, and its silencing could increase cell sensitivity to 5FU. Moreover, MINK1 negatively regulated p53 in 5FU-resistant OSCC through the AKT/MDM2 axis. Mohanty et al. suggested that MINK1 could be used as a therapeutic target for 5FU-resistant OSCC. After the evaluation of the data from the International Union of Basic and Clinical Pharmacology (IUPHAR) database, staurosporine, pexmetinib, and lestaurtinib were tested for MINK1 inhibition. Lestaurtinib and pexmetinib turned out to have the highest MINK1 inhibitory activity according to kinase assay data. Importantly, they could restore 5FU sensitivity in resistant cells, but not in cells depleted from MINK1, which highlights the fact that the effect of decreased 5FU resistance resulted from MINK1 inhibition. A significantly reduced relative number of migrating cells was observed after combinatorial treatment with lestauritinib and 5FU, while the combinatorial group of cisplatin, 5FU, and lestauritinib exhibited significantly higher cell death compared to any other combination. This indicates that targeting MINK1 could be beneficial for OSCC patients resistant to current chemotherapy. It is possible, that MINK1 inhibition will bring satisfactory results in other malignancies as well, and for this reason, studies on the topic are of high importance. Lestaurtinib has been tested for AML treatment, but no positive results were observed [164]. The failure of lestaurtinib in AML highlights the need for cancer-specific studies to fully understand the therapeutic potential of MINK1 inhibitors. Discussed research highlight MINK1 as a potential therapeutic target to overcome 5FU resistance, which could significantly improve treatment outcomes for OSCC patients. Pexmetinib is a p38 and Tie2 kinase inhibitor with potential use in myelodysplastic syndrome and arthritis, and it is currently being investigated in a clinical trial [National Center for Biotechnology Information. PubChem Compound Summary for CID 24765037, Pexmetinib. https://pubchem.ncbi.nlm.nih.gov/compound/Pexmetinib, accessed on 7 March 2024]. Nevertheless, future discoveries may bring novel options for MINK1 targeting. Exploring the therapeutic potential of MINK1 in various cancer types could generate new hopes for treatment, especially in the event of drug resistance.

### 5.3. Colorectal Cancer

MINK1 has been also implicated in colorectal cancer (CRC). Adenomatous polyposis coli (APC) is the most commonly mutated gene in colorectal cancer and was shown to be involved in Wnt canonical signaling [24,165]. Popow et al. showed that MINK1 can interact with both WT and truncated APC expressed in CRC [24]. APC silencing resulted in a concomitant increase in MINK1 levels both in vitro and in vivo. However, the changes in MINK1 protein abundance did not correlate with mRNA, suggesting a posttranscriptional mechanism of regulation. These findings suggest that targeting posttranscriptional mechanisms regulating MINK1 could offer a novel therapeutic approach in CRC. MINK1 was downregulated by APC independently of Wnt-caconical signaling. In addition, an increase in cell–cell adhesion to collagen was observed in the event of MINK1 overexpression. Importantly, when MINK1 was depleted using siRNA, there was a significant reduction in the proliferation of colorectal cancer cells in which MINK1 regulation by APC was lost. These results indicate that MINK1 could serve as a therapeutic target in CRC, but more data on the topic are needed [24]. MINK1 may take part in tumorigenesis via other signaling pathways as well, thus its function in colorectal cancer can be multi-dimensional. It is also necessary to establish the influence of MINK1 knockdown on cell adhesion and clinicopathological factors in the disease.

### 5.4. Other Malignancies

MINK1 has been also implicated in the progression and prognosis of other malignancies, including glioblastoma (GBM), hepatocellular carcinoma (HCC), and head and neck squamous cell carcinoma (HNSC).

McInerney et al. found that MINK1 expression was significantly downregulated in glioblastoma cells compared to non-tumor tissue (*p* < 0.001) [166]. In addition, MINK1 expression was negatively associated with the expression of isocitrate dehydrogenase (IDH) 1. IDH1 overexpression in GBM has been connected to disease progression and therapy resistance, while its genetic and pharmacological blockage could improve clinicopathological conditions and response to therapies during the disease [167]. It is thus possible that MINK1 could be used for determining treatment options for GBM patients, while targeting pathways related to it might influence IDH1 expression, being a possible therapeutic strategy.

MINK1, along with NF2, MYC, BIRC3, and CSNK1E, was also included in a prognostic model for hepatocellular carcinoma based on the expression of Hippo-related genes (HRGs) in adjacent tissues [18]. Notably, the risk score calculated this way showed better predictive value (AUC = 0.750) than clinicopathological parameters, including tumor stage (AUC = 0.735). This indicates that MINK1 could play an important role in HCC, which is yet to be established.

The prognostic role of MINK1 was also demonstrated in another study. Zhang et al. aimed to study correlations between the dysregulation of mRNA and long non-coding RNAs (lncRNAs) and the survival in head and neck squamous cell carcinoma [19]. The authors found mRNAs and lncRNAs to be related to the HNSC survival in three datasets and selected seven gene signatures for further assessment of the risk score model. Among the selected mRNAs, MINK1 mRNA was detected as altered in tumor tissues in comparison to the normal tissue samples. Its hazard ratio was marked as negative, which indicated that MINK1 might be a tumor suppressor gene. However, the calculated *p*-value for MINK1 was higher than in the case of other detected genes (*p* = 0.05), thus the relevance of the role of MINK1 may be questioned. Meanwhile, evaluated simultaneously with other selected genes (LCLAT1, WDTC1, TOM1L2, AMPD3, CCDC43 mRNAs, and one lncRNA: RAB11B-AS1), MINK1 was identified as a valuable prognostic marker. The risk score was not dependent on clinicopathological features, and the authors stated that it was a better prognostic of the survival in HNSC than clinical information, as it assessed successfully the survival of 755 HNSC samples in two platforms and in five datasets. This means that MINK1 may take part in HNSC pathogenesis.

As mentioned before, RAS signaling plays an important role in oncogenesis, and RAS genes are often mutated in tumors [55,56,57]. Nicke et al. uncovered that oncogenic Ras can induce growth arrest in human ovarian surface epithelial cells and that the process is dependent on MINK1 [4]. Ras induction managed to increase MINK expression by 2-fold, likely via the Raf/MEK/ERK pathway; MINK then elevated p38 MAPK activity and p21^WAF1/CIP1^ levels while decreasing cyclin A, contributing to the growth arrest induction. Moreover, MINK may be involved in this process in other cell populations as well. Still, 43% ± 6% of cells with hushed MINK were able to overcome the growth arrest in the event of oncogenic Ras expression. However, the authors pointed out that this could be due to poor MINK knockdown, possibly caused by its heterogeneity—26% of MINK expression was detected in MINK-hushed cells compared to controls. Notably, there is no evidence that MINK influences Ras-induced transformation in cancer cells. MINK1 induction could be considered a therapeutic strategy for ovarian cancer for induction of growth arrest in tumor cells. However, its function in the process could be influenced by TNIK. These results offer a novel perspective on MINK1 function in tumors, but they need confirmation in future reports.

**Figure 8:**
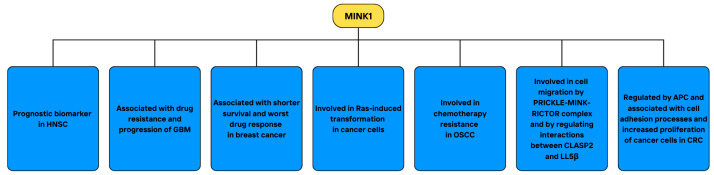
MINK1 as a key component in different cancer types. HNSC—head and neck squamous cell carcinoma; GBM—glioblastoma multiforme; OSCC—oral squamous cell carcinoma; PRICKLE—Prickle planar cell polarity protein; MINK1—Misshapen-like kinase 1; RICTOR—rapamycin-insensitive companion of mTOR; CLASP2—cytoplasmic linker-associated protein 2; LL5β—pleckstrin homology domain-containing family L member 5β; APC—adenomatous polyposis coli; and CRC—colorectal cancer.

Details about current research considering the role of MINK1 in cancer, including its altered expression, associations with prognosis, and related patomechanisms, are demonstrated in Figure 8. In summary, there is still a lot to uncover regarding the role of MINK1 in oncogenesis. Its range of functions is likely broad, and more studies are needed to elucidate its therapeutic potential in cancer. The role of MINK1 in chemotherapy resistance seems to be especially promising. Novel strategies of MINK1 inhibition in drug-resistant patients could be used to restore their sensitivity to currently available therapies and to improve their prognosis. Targeting MINK1 might also influence the clinicopathological features of cancer patients, reducing cancer cell tendencies for metastasis. Nevertheless, the data on the subject are still limited and the function of MINK1 in cancer requires further investigation.

## 6. The Role of MINK1 in Other Systems and Pathological Conditions

Studies concerning MINK1 expression and function have also been beneficial for exploring the nature of various other pathological conditions, including the cardiovascular, nervous, skeletal system, and others. The results may contribute to deepening the understanding of diseases with unclear pathogenesis and give hope for novel and more effective treatment pathways in the future.

### 6.1. Cardiovascular System and Hemostasis

CVS whole-exome sequencing by Jin et al. revealed de novo mutations (DNMs) related to congenital heart disease (CHD) in 12 new genes. Among them, mutations in MINK1 reached genome-wide significance [2]. CHD is one of the most common birth defects and it is suggested that its genetic causes can be determined in 20 to 30% of cases [168,169,170]. Studies addressing the genetics of congenital heart disease are highly important and could contribute to determining risk factors, predicting the course of the disease, and providing patients with improved clinical care.

The role of MINK1 in CHD was investigated by Colleluori and Khokha [171]. Utilizing *Xenopus tropicalis* embryos, they demonstrated that MINK1 depletion increases the chances of impaired heart development. MINK1-depleted tadpoles showed significantly more incidences of abnormal outflow tract (OFT) looping, remaining unlooped or looping to the left instead of to the right. Similar effects were caused by MINK1 overexpression and were likely dependent on MINK1 kinase activity. MINK1 knockdown also led to abnormalities in left–right (LR) axis patterning, as shown by altered expression of LR patterning markers, pitx2c and dand5 [172], as well as a higher rate of improper gastrulation. The results suggest that MINK1 is required for proper left–right organizer (LRO) patterning in embryos, and may affect Spemann organizer cell fate, as demonstrated by the altered and reduced expression of markers foxj1, goosecoid, and nodal3 in the absence of MINK1. Moreover, Spemann’s organizer cell fate is dependent on the Wnt canonical pathway and β-catenin is essential for its function [173]. MINK1 is known for its inhibition of this signaling, as explained earlier in the text [16]. The authors showed that MINK1 loss leads to the loss of β-catenin protein and that MINK1 functions upstream from it in the Wnt canonical pathway [171]. Since β-catenin injection could significantly rescue the abnormal pitx2c expression in MINK1 crispants, the authors concluded that the defects in LR patterning were caused by the misregulation of Spemann organizer cell fate. This was likely the result of abnormalities in β-catenin-dependent Wnt signaling. Moreover, quantitative phosphoproteomic analysis revealed HMGA2 as a possible candidate for downstream MINK1 effector in this pathway. HMGA2 is a chromatin-associated transcription factor and has been shown to play an essential role in cardiogenesis in Xenopus [174,175]. Another study revealed that off-target MINK1 inhibition could be responsible for cardiovascular malformations in rats treated with an antimalarial candidate MMV390048 [176], which further highlights the importance of MINK1 in the development of the cardiovascular system.

The role of MINK1 in blood hemostasis is an interesting area for investigation as well. Yue et al. demonstrated that MINK1 can promote hemostasis and thrombosis in vivo by regulating platelet dense granule secretion [177]. Dense granules contain calcium, magnesium, ATP, ADP, 5-hydroxytryptamine (5-HT), and other small molecules that ensure proper platelet aggregation, activation process, and thrombus formation [79,178,179]. MAPKs and PI3K/Akt pathways have been reported to play a role in the regulation of platelet secretion [180,181,182,183], and MINK1 has been found to take part in the activation of MAPKs such as p38 and JNK [4,7,123,153]. Still, little is known about the complex molecular networks regulating platelet function.

**Figure 9:**
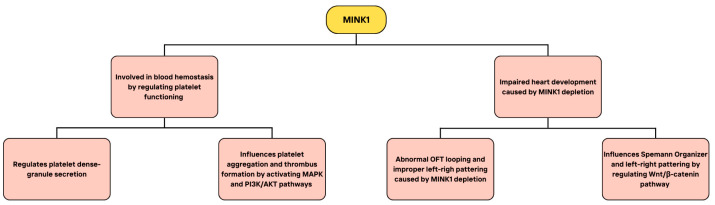
MINK1 in cardiovascular diseases. MINK1—Misshapen-like kinase 1; MAPK—mitogen-activated protein kinase; PI3K—phosphoinositide 3-kinase; AKT—AKT serine/threonine kinase; and OFT—outflow tract.

The study performed by Yue and colleagues was conducted on MINK1-deficient (MINK1^−/−^) mice, which exhibited a longer time of bleeding in the tail bleeding assays in comparison to wild-type mice [177]. The authors pointed out though, that it could have been caused by possible defects of blood vessels upon MINK1 deficiency. The MINK1^−/−^ mice also showed twice as long vessel occlusion time in a model of ferric chloride-induced mesenteric arteriolar thrombosis. Moreover, MINK1^−/−^ platelets demonstrated significantly reduced thrombus formation on a collagen matrix in an in vitro microfluidic whole-blood perfusion assay. Thrombi formed by MINK1-deficient platelets were significantly smaller than those formed by wild-type platelets. MINK1^−/−^ platelets also showed dysfunction of aggregation and dense granule secretion in reaction to thrombin and collagen in low doses. The latter effect could not be reversed by the treatment with higher concentrations of collagen, thrombin, and U46619. WT platelets exhibited similar defects of aggregation in the presence of apyrase which hydrolyzed adenosine 5′-diphosphate (ADP). At the same time, ADP supplementation in low doses could rescue the impaired aggregation and spreading on fibrinogen of MINK1^−/−^ platelets. The authors therefore suggested that these defects may occur due to impaired ADP secretion despite normal levels of ADP in MINK1-deficient platelets. MINK1^−/−^ platelets showed decreased levels of phosphorylated extracellular signal-regulated kinase (ERK), p38, and Akt in response to thrombin and collagen, even in comparison to WT platelets treated with apyrase. This may indicate that MINK1 could regulate these phosphorylation events not only via reduced ADP secretion, but also independently of it. The authors also confirmed that the decrease in ERK and p38 phosphorylation is responsible for the disrupted aggregation of MINK1^−/−^ platelets. The data show the importance of MINK1 in the regulation of dense granule secretion in reaction to collagen and thrombin, as well as its essential role in mitogen-activated protein kinase signaling and PI3K/Akt pathway. These results demonstrate that MINK1 deficiency impairs platelet function by disrupting ADP secretion and MAPK/PI3K-Akt signaling, emphasizing its critical role in hemostasis. The study provided useful information for further investigating the process of platelet dense granule secretion and consequently the nature of various hematological diseases. The summary of the presented studies on the role of MINK1 in the cardiovascular diseases, along with the respective processes it regulates, including the function in hemostasis and heart development, is illustrated in Figure 9.

### 6.2. Nervous System and Neurodegenerative Diseases

Several studies show that MINK1 is expressed mainly in brain tissue [7,23], so its function in the nervous system has been an area of interest for many researchers. MINK, along with TNIK, is abundantly present in synapses and is an important component of postsynaptic density (PSD) [23,184]. Normally, MINK1 interacts with Rap2 in the brain and Rap2 enhances MINK1 kinase activity [15], while Rap2-JNK pathway contributes to the removal of AMPA receptors (AMPA-Rs) during depotentiation [185]. Hussain et al. demonstrated that altered MINK, which is unable to interact with Rap2, results in the atrophy of dendritic arbors, and that MINK overexpression leads to the disruption in the removal of AMPA-Rs mediated by Rap2 [23]. These results suggest that MINK negatively regulates Rap2-mediated signaling, influencing AMPA-R dynamics and neuronal structure.

Targeting MINK resulted in a major decrease in dendritic arbor complexity in comparison to control neurons, and a similar effect was observed upon TNIK knockdown. Silencing either MINK or TNIK led to lower density of dendritic spines and resulted in decreased density of both surface GluR1 (sGluR1) and surface GluR2 (sGluR2) dendritic clusters in hippocampal dendrites. MINK targeting reduced significantly the amplitude of AMPA-EPSCs (excitatory postsynaptic currents) relative to nontransfected neurons, showing the essential role of MINK in surface AMPA-R function and expression. Hussain et al. also showed that MINK likely interacts with Rap2 via its CNH domain in hippocampal neurons independently from its kinase activity. Moreover, Rap2 modulates the distribution of both MINK and TNIK, which is dependent on its CAAX motif. The active Rap2 is also required for the reduction in dendrite complexity, which occurs in the presence of truncated MINK, lacking its CNH domain. The authors concluded that MINK prevents the Rap2-mediated loss of surface AMPA-Rs. The disruption of the interactions between MINK and Rap2 abolishes the restriction of Rap2 signaling, resulting in altered structure and function of neurons. It is hence possible that MINK1 takes part in the pathogenesis of various diseases, in which neuronal morphology is altered, and which include mental retardation, epilepsy, schizophrenia, and Alzheimer’s disease [186,187,188].

Indeed, according to existing reports, MINK1 seems to play a role in neurodegeneration and more neuronal functions. Larhammar et al. found the Ste20 kinases MAP4K4, MINK1, and TNIK to regulate DLK/JNK signaling in neurons [3]. Dual leucine zipper kinase (DLK) is necessary for axonal degeneration induced by growth factor deprivation. It contributes to the c-Jun N-terminal kinase (JNK) phosphorylation and the transmission of the stress signal to the nucleus [75]. In a trophic factor withdrawal-based neurodegeneration model in ganglion neurons of embryonic mouse dorsal root, the kinases MAP4K4, MINK1, and TNIK had redundant effects leading to DLK regulation and stress-induced c-Jun phosphorylation [3]. The authors demonstrated that inhibiting these kinases simultaneously protects neurons from degeneration. The results highlight the importance of MAP4Ks, including MINK1, in the regulation of the DLK/JNK signaling pathway.

The authors found that MAP4K4 inhibitors have effective neuroprotective properties, but these results may be partly due to the homology of the MAP4K4 kinase domain and the kinase domain of MINK1 and TNIK [7]. Moreover, the loss of MAP4K4 expression was not sufficient to compromise the activation of DLK and c-Jun phosphorylation in the event of NGF withdrawal, but neither was MINK1 or TNIK targeting. Only the knockdown of all three kinases resulted in decreased p-JNK and p-c-Jun induction in NGF-deprived neurons and protection against axonal degeneration. DLK was shown as well to be necessary for maintaining c-Jun phosphorylation and DLK inhibitor could reverse c-Jun phosphorylation completely. On the other hand, MAP4K inhibitor could prevent c-Jun phosphorylation when added shortly after NGF withdrawal, but did not influence p-c Jun levels when administered three hours later. This suggests that MAP4K4, TNIK, and MINK1 may initiate or propagate DLK/JNK retrograde signaling, but are not required for maintaining DLK activation. Importantly, the removal of NGF from distal axons was sufficient to trigger c-Jun phosphorylation, and consistently, the addition of MAP4K inhibitor to axons resulted in the inhibition of p-c Jun induction. Together, these results show that MAP4Ks, including MINK1, act in the axon to regulate retrograde DLK/JNK signaling, contributing to axonal degeneration following NGF deprivation.

As the JNK signaling pathway is a key regulator in the development of the nervous system, as well as axon regeneration and neuronal degeneration following injuries and chronic neurodegenerative diseases [64,75,189,190], the authors presented MINK1, MAP4K4, and TNIK as potential therapeutic targets, although MAP4K4 and TNIK may be responsible for the majority of the presented effects.

As mentioned, MINK1 takes part in synaptic morphology and neural degeneration, so it could be involved in the pathogenesis of Alzheimer’s disease (AD) [3,23]. AD is a leading cause of dementia and is quickly becoming one of the most concerning diseases in the modern world [191]. It is a disorder characterized by the presence of amyloid β and tau protein phosphorylation, accompanied by progressing memory impairment [192,193,194]. Numerous phosphopeptides are altered in the brains of patients with Alzheimer’s disease, as revealed by analyses of postmortem human brain tissues, suggesting that kinases, including MINK1, may play a role in the pathogenesis of AD [195].

More studies shed some light on this issue. Utilizing genome-wide association studies (GWAS) summary statistics of the International Genomics of Alzheimer’s Disease Project (IGAP Stage 1 and 2), Broce et al. evaluated the data to elicit potentially useful correlations between cardiovascular (CV)-associated genes and Alzheimer’s disease (AD) [196]. The authors found 90 SNPs on 19 chromosomes, including four novel variants, which together seemed to increase the risk of AD in CV outcomes. Among them, DDB2, MBLAC, and MINK1 were associated with proxy AD status in the UK Biobank sample. The authors identified a MINK1 variant on chromosome 17, and its expression to be altered in AD brains. Broce and colleagues showed that various CV-associated risk factors were connected to an increase in the risk of AD, even for people with genetic predispositions for developing Alzheimer’s disease, proving the complex nature of the disease and the possible role of MINK1 in its development. However, it was highlighted that no single variant identified in the study can be clinically informative, and only integration of the pleiotropic variants may help identify individuals at risk of both CV and Alzheimer’s disease. Nevertheless, CV factors, including MINK1, may serve as a potential therapeutic target, as that could impact the trajectory of AD.

**Figure 10:**
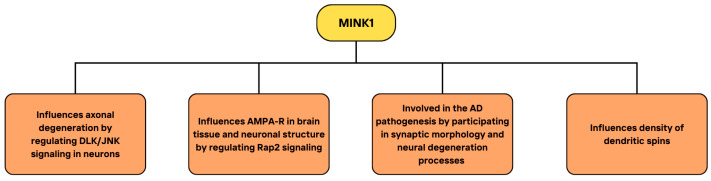
MINK1 role in the nervous system and in neurodegenerative diseases. DLK—dual leucine zipper kinase; JNK—c-Jun N-terminal kinase; AMPA-R—alpha-amino-3-hydroxy-5-methyl-4-isoxazolepropionic acid receptor; AD—Alzheimer’s disease; and MINK1—Misshapen-like kinase 1.

There is more evidence supporting the claim that MINK1 takes part in AD onset and may be clinically useful for patients at risk of developing AD. Lawingco et al. assessed the contribution of polygenic risk score (PRS) consisting solely of genes encoding synapse-related proteins in predicting late-onset Alzheimer’s disease (LOAD) [197]. Although the contribution of MINK1 to the model is relatively low (5%), together with seven other variants, it helps in evaluating the risk of LOAD with high accuracy. The synaptic PRS model including MINK1 is the simplest model allowing for identifying patients at risk of Alzheimer’s disease before the occurrence of first symptoms. However, the results should be cautiously approached and confirmed in future studies. The role of MINK1 and other polymorphisms mentioned in the study should be further evaluated in the context of AD. The summary of the role of MINK1 in the nervous system and in neurodegenerative disorders, including MINK interactions with relevant proteins and signaling pathways, is demonstrated in Figure 10.

### 6.3. Other Systems and Conditions

The role of MINK1 is not restricted to previously mentioned diseases, but can be explored in much more depth. Another condition with pathogenesis that could be dependent on MINK1 is osteoarthritis, the most common disease of the joints. This progressive condition, which eventually can lead to the loss of joint function, is characterized by articular cartilage degeneration, synovial inflammation and fibrosis, formation of osteophytes, and alterations of subchondrial bone [198,199]. All these processes are influenced by the MINK1 function. Yu et al. showed that MINK1 is expressed in human cartilage, and this expression is decreased in patients with osteoarthritis (OA) compared to healthy cartilage [200]. The authors described the abundant expression of the protein during the development of mouse joints and suggested that MINK1 may take part in chondrocyte hypertrophy. MINK1 knockdown, however, did not affect the length of mouse limbs, nor the skeleton development. Importantly, MINK1 was only present in the deep layer of adult cartilage affected by the OA, while in the healthy articular cartilage, it could also be detected superficially, suggesting that MINK1 may be involved in OA pathogenesis. MINK1 deletion in OA was shown to decrease the degeneration level, and aggrecan and type II collagen degradation in the surface level of 12-month cartilage compared to WT mice in an age-related OA model. The primary chondrocytes of MINK1-deficient mice exhibited elevated levels of type II collagen synthesis as well. On the contrary, MINK1-depleted mice suffered from more cartilage destruction in injury-induced OA. This could be caused by the destabilization of not only the cartilage, but also the whole joint. MINK1^−/−^ mice showed also an increased tendency for chondrophyte formation, which could be decreased via injection with a bone morphogenetic protein (BMP) receptor inhibitor. Additionally, the activity of alkaline phosphatase, as well as calcium deposition, was enhanced in MINK1^−/−^ chondrocytes. Nestin-positive mesenchymal stem cells (MSCs) and osterix-positive osteoprogenitors were also detected at higher levels in the bone marrow of MINK1^−/−^ compared to wild-type animals.

The bone morphology was significantly altered in MINK1^−/−^ mice, with increased bone mass, exacerbated subchondral bone sclerosis, and subchondral bone plate (SBP) penetration into cartilage. Furthermore, Yu and colleagues detected increased SMAD2 phosphorylation in MINK1^−/−^ mice, which was reduced by MINK1 overexpression. The authors suggested that MINK1 inhibits the phosphorylation process of SMAD2 in MSCs in the bone marrow, exacerbates the subchondral bone morphology alterations, and contributes to the aggravation of OA induced by injury. MINK1 has been already recognized as the suppressor of TGF-β/BMP signaling, likely inhibiting the C-terminal phosphorylation of SMAD2 resulting from its interactions with TGF-β/BMP receptor kinase. These interactions may be altered in the case of SMAD phosphorylation by MINK1, explaining lowered levels of pSMAD2 detected by Yu et al. in the presence of MINK1 [134,201]. MINK1 inhibitors could probably be utilized to delay OA onset, but the exact role of MINK1 in OA should be clarified first.

As mentioned before, MINK1 plays an important role in the cell cycle and cytoskeleton organization [10,99], while mechanical forces have long been known to influence cell behaviors [202]. Hang et al. showed that heterogeneities in mechanical properties between drug-resistant and drug-sensitive cells can be detected and may depend on differences in gene expression [163]. Heterogeneities cause cells to react differently to exogenous stimulation, and dysregulation of mechanical forces applied to cells may result in tendencies for tumorigenesis, metastasis, or drug resistance [203,204,205,206,207,208]. In the study, drug-resistant cells exhibited elevated expression of MINK1, and its silencing resulted in lowered mechanical forces of the cells [163]. The results imply that the MINK1 gene takes part in the regulation of cell mechanical forces, contributing to the occurrence of drug resistance. Tracking the expression of MINK1 may be useful in the identification of potential drug resistance in tumor cells. Moreover, mechanical forces differ between metastatic and non-metastatic cells, with the former showing an increase in mechanical force and higher MINK1 levels. This further confirms the potential importance of MINK1 expression assessment in tumor cells.

Additionally, MINK1 was shown to take part in vasopressin signaling in the renal collecting duct. Vasopressin regulates the osmotic water permeability in principal cells of the collecting duct by controlling the trafficking of vesicles containing a water channel aquaporin-2 (AQP2), via its phosphorylation at Ser^256^, and enhancing the rate of the transcription of the Aqp2 gene [209,210,211,212]. Park et al. aimed to identify new sites of vasopressin-induced phosphorylation in the collecting duct in rat and mouse models to detect proteins regulating AQP2 dynamics for future studies on their function [213]. The authors arranged the data on significant changes in phosphorylation sites after treatment with V2 receptor-specific vasopressin analog desmopressin, 1-deamino-8-D-arginine vasopressin (dDAVP), in cultured collecting duct cells from the mouse (mpkCCD) and native rat inner medullary collecting duct (IMCD) cells into one data set. Park and colleagues used an automated bioinformatic extractor (ABE) and UniProt to elicit information on detected proteins and their gene symbols. They identified 45 proteins and 51 phosphorylation sites that demonstrated substantial changes both in cultured mouse mpkCCD cells and native rat IMCD suspensions in response to the vasopressin analog [214]. MINK1 phosphorylation decreased upon treatment with dDAVP, and it was suggested to act downstream from PKA. Its exact role in the collecting duct in response to vasopressin requires, however, further investigation. Still, little is known about proteins that undergo phosphorylation mediated by vasopressin, and more studies in this area could reveal useful information about the function of the renal collecting duct—including the specific function of MINK1.

It is also worth mentioning a case study of an individual with a de novo balanced reciprocal translocation: t(17;19) (p13;p11), with the first translocation breakpoint located on 17p13, between exon 1 and exon 2 of MINK1, and the second in the chromosome 19 centromere [215], that could illustrate a versatile role of MINK1 in the proper development of various systems of the human body. The patient had an inborn cataract of the left eye and suffered from depression, autism, osteoporosis, and epilepsy. The translocation had an impact on the expression of 539 genes in disease-relevant patient-specific neural cells, with MINK1 expression being significantly decreased in comparison to healthy cells. Moreover, 70% (376 of 539) of the genes with altered expression belonged to the network of MINK1 interactors. The authors hypothesized that the disruption of MINK1 expression could be responsible for the patient’s clinical state. Considering that MINK1 has been implied to play a role in a variety of pathological conditions in humans [1,76,177,197,200], it would be beneficial to further test its influence in conditions present in the case of the patient: autism, congenital cataracts, epilepsy, and osteoporosis.

Many more conditions were reported as possibly related to the MINK1 gene polymorphisms. Outside of previously mentioned diseases, the genome-wide association study (GWAS) database reveals SNPs in MINK1 amyotrophic lateral sclerosis, bipolar disorder, astigmatism, breast neoplasms, prostatic neoplasms, Crohn’s disease, insulin resistance, Parkinson’s disease, Hodgkin disease, schizophrenia, and type II diabetes (GWAS Central at www.gwascentral.org, accessed on 28 November 2024 [216]), which is a significant foundation for future studies.

## 7. Summary and Conclusions

Misshapen/NIKs-related kinase (MINK) 1 is a relatively novel subject of research in the field of molecular biology, and the data regarding its function are limited. Even less is known about MINK1’s role in clinical conditions. In this review, we attempt to summarize the information available on MINK1, to highlight its potential applicability in clinical practice, and to encourage a broader investigation of its role in various pathological states.

MINK1 is a meaningful component in many cellular signaling pathways. Through its role in the β-catenin-independent WNT signaling, it takes part in the regulation of not only planar cell polarity and convergent extension during embryogenesis, but also cell motility [9,16]. Acting downstream of Dishelved, it can modulate the activity of Prickle. MINK1 has been also shown to form complexes with Prickle1 and RICTOR [43]. It interacts with Ras proteins and is involved in Ras-induced growth arrest—this involvement likely engages the Raf/MEK/ERK pathway and is dependent on ROS [4]. This results in the modulation of p38 MAPK activity, but it requires further studies to determine how exactly MINK1 controls p38. MAP3K5 (Ask1) has been suggested as a link connecting the activity of these two kinases, and this area could be explored in the future. Moreover, MINK1 interacts with Rap2, and under its control, it induces phosphorylation of a postsynaptic density protein TANC1 [15]. These interactions would also be interesting targets for further investigations. Additionally, MINK1 acts upstream from JNK to promote its activation [73] and could induce the phosphorylation of LATS and YAP in the Hippo signaling [97]. Novel reports introduce MINK1 as a STRIPAK component [17,99]. Indeed, it has been demonstrated to immunoprecipitate with STRN4 (Zinedin) and PPP2CA, and to form complexes with FAM40A/B (STRIP1/2), SLMAP, and CTTNBP2NL [99]. Still, little is known about the role of MINK1 in STRIPAK, and future studies could shed more light on this area. Notably, along with Zinedin, MINK1 could regulate cell division by modulating abscission, the final stage of cytokinesis. Clarifying the interactions of MINK1 with the mentioned proteins and elucidating its role in the named pathways could provide novel information on the role of MINK in cancerogenesis and cancer progression, and as a result, contribute to developing new therapeutic strategies in malignancies.

Interesting reports exist concerning the role of MINK1 in immunological processes and diseases. MINK1 expression is boosted in the presence of ROS, which is crucial in the case of numerous inflammatory responses [4,126]. MINK1 regulation by ROS may explain the effects of ROS on Th17 cell differentiation, as MINK1 suppresses their development [126]. These results reveal as well the complexity of the redundant influence of reactive oxygen species on cells. Still, a more concrete mechanism of MINK1 regulation by ROS remains to be elucidated. Additionally, the negative regulation of Th17 cells appears to take place through SMAD2 phosphorylation. SMAD2/MINK interactions could be another area for future studies, and the results could be useful in understanding and alleviating Th17-driven inflammatory processes. MINK1 also takes part in NLRP3 inflammasome activation [125], which suggests its role in related diseases, such as type 2 diabetes, gout, and sepsis [138,139,140]. Other reports implicate MINK1 in rheumatoid arthritis, asthma, and SLE [1,19,123]. Considering the complex network of MINK1 interactions, its relations to essential signaling pathways, and ROS levels, there certainly is a lot more to discover in the area of MINK1 in immunity. Autoimmune diseases could be investigated in this context since little is known about their pathogenesis. Inducting or targeting MINK1 could serve as a therapeutic strategy in such conditions. The interplay of the kinase with MBD2 could be further studied as well, especially in the conditions involving Th17 cells [1]. Moreover, miR-17-5p seems to target MINK1, and SNHG14 counterplays this regulation in rheumatoid arthritis [76]. Since miR-17-5p has been implicated in neuronal impairment in cerebral ischemia, its interactions with MINK1 could be explored in this context too. Interestingly, MINK1 enables EV71 replication and could take part in the replication of other enteroviruses [153]; hence, it would be beneficial to study MINK1 as a potential antiviral target.

MINK1 is involved in the pathogenesis of various malignancies. It is downregulated in glioblastoma [166], while in breast cancer, its upregulation is connected to the worse overall survival of the patients [17]. MINK1 expression was included in prognostic models for head and neck squamous cell carcinoma (HNSC) and hepatocellular carcinoma (HCC), being marked as a tumor suppressor gene for the former [18,19]. In addition, MINK1 downregulated IDH1, which is associated with bad prognosis in glioblastoma [166,167]. MINK1 silencing resulted in decreased motility and proliferation of breast cancer and CRC cells, as well as reduced viability of OSCC cells [24,43,162], it can thus be involved in the metastatic spread of cancer cells and its targeting may be a good treatment strategy for several types of cancer. Moreover, MINK1 was shown to interact with APC in colorectal cancer, which is an interesting direction for future investigations [24]. MINK1 likely mediates a Ras-induced growth arrest in cancer cells as well [4]. Notably, the existing reports suggest that MINK1 may be a key factor responsible for drug resistance in cancer patients. 5FU insensitivity in OSCC is mediated by MINK1, while its silencing managed to abolish this resistance [162]. Targeting MINK1 in such cases is a promising strategy for patients exhibiting resistance to chemotherapy. Overall, MINK1 probably plays a complex role in cancer, acting as a tumor suppressor, or contrarily, as a factor promoting aggressive phenotype of cancer cells depending on the case. Unfortunately, little is known about its role in this context, and elucidating more data on MINK1 function in cancer should be a goal for future studies.

MINK1 has been implicated in several other conditions. MINK1 contributes to the development of congenital heart disease and takes part in heart development, regulating Spemann organizer cell fate possibly via its role in the inhibition of canonical Wnt signaling and through HMGA2 protein [2,171]. Further investigation would be needed to answer the questions of how exactly HMGA2 relates to the Wnt signaling and how it interacts with MINK1. A more detailed role of MINK1 in Speman organizer and β-catenin protein level regulation needs to be established as well. It is also not clear whether MINK1 directly phosphorylates HMGA2—it most likely does not, so it would be useful to pinpoint direct MINK1 targets or the kinases responsible for direct HMGA2 phosphorylation. Moreover, MINK1 takes part in thrombosis regulating the function of p38 and ERK, but the complex network of its interactions in hemostasis-related processes remains unclear [177]. It should be further investigated which molecules are activated by MINK1 downstream of MAPKs. It could be also beneficial to examine the role of MINK1 in hematological and cardiovascular diseases in more detail, not only in animal models, but in humans as well.

As MINK1 is an important component of neuronal and synaptic structure, it would be crucial to investigate whether more neuronal processes could depend on MINK [23]. The specific interactions between MINK and Rap2 leading to different outcomes in neurons should also be studied. Since MINK has been demonstrated to regulate synaptic morphology, it could potentially play a role in conditions in which this morphology is altered, such as mental retardation, epilepsy, schizophrenia, and Alzheimer’s disease [186,187,188]. Indeed, MINK1 is altered in AD, and it could be taken into consideration as a potential therapeutic target or prognostic marker in the disease [196]. MINK1 plays a role in neurodegeneration via DLK regulation in JNK signaling; however, detailed mechanisms behind these phenomena are not explained [3]. Influencing MINK1 expression and function in neurodegenerative diseases could serve as an additional treatment strategy and it would be interesting to explore the therapeutic potential of MINK in neurology. Still, it remains uncertain how exactly the MAP4Ks are activated in mammalian neurons, and this question requires further investigation. It is unclear how MAP4Ks regulate the DLK/JNK pathway either. In AD, the phosphorylation events also involve heat shock proteins (HSPs). Investigating the crosstalk between MINK1 and various HSPs could reveal more useful information with potential clinical significance [195].

Since MINK1 seems to play a role in osteoarthritis development [200], MINK1 inhibitors could be used to delay OA development and reverse the changes in SMAD2 signaling related to age, but it would be necessary to clarify if MINK1 is responsible for the downfall in SMAD2 phosphorylation or if there are other factors, for example, increased severity of OA in MINK1^−/−^ mice in general, responsible for this effect. MINK1 also takes part in the regulation of cell mechanical forces, which vary between metastatic and non-metastatic tumor cells, as well as between drug-resistant and drug-sensitive cells [163], which is another reason why it would require further investigation of whether MINK1 expression is altered in human neoplasms and whether the measurement of its levels could contribute to establishing more effective clinical pathways for patients. Interestingly, MINK1 also undergoes phosphorylation changes in the renal collecting duct in response to vasopressin [213], and identifying its specific role, kinase function, and interactions with other proteins in the collecting duct would be an interesting direction for further studies.

According to GWAS [216], polymorphisms in the MINK1 gene have been detected in many more conditions, including epilepsy, amyotrophic lateral sclerosis, bipolar disorder, astigmatism, breast and prostatic neoplasms, Crohn’s disease, insulin resistance, Parkinson’s disease, Hodgkin disease, schizophrenia, or type II diabetes. Inducing or inhibiting MINK1 in such conditions, as well as determining the factors influencing MINK expression, could alleviate the course of diseases. However, there are still many challenges and knowledge gaps that further research should address. The dual roles of MINK1 as both a tumor suppressor and a promoter of aggressive cancer phenotypes suggest that the function of MINK1 may depend on various factors and may be different in various cancers. Further studies are needed to understand how environmental factors, such as the tumor microenvironment and the infiltration of the immune cells, may influence MINK1’s role in various cancers. There is also a need for more studies examining the clinical implications of targeting MINK1 in therapies, particularly in cancers and autoimmune diseases. Understanding how MINK1 inhibition or activation affects patient outcomes could provide insights for therapeutic strategies. Most of the findings are cross-sectional, indicating a need for longitudinal studies to assess how MINK1 expression and function change over time in relation to disease progression. The potential influence of genetic polymorphisms in the MINK1 gene, and how post-translational modifications of MINK1, such as phosphorylation or ubiquitination, affect its function, could significantly enhance our understanding of MINK1’s roles in cellular signaling and its implications in various diseases. We sincerely hope that future studies will bring new insights into the mystery surrounding misshapen/NIKs-related kinase 1 and the complex networks of its interactions, revealing new prognostic markers or possible therapeutic pathways.
